# Healthy Aging in Times of Extreme Temperatures: Biomedical Approaches

**DOI:** 10.14336/AD.2023.0619

**Published:** 2024-04-01

**Authors:** Axel Kowald, Daniel Palmer, Riccardo Secci, Georg Fuellen

**Affiliations:** ^1^Institut für Biostatistik und Informatik in Medizin und Alternsforschung, Universitätsmedizin Rostock, Germany.; ^2^Interdisziplinäre Fakultät, Department AGIS (Altern des Individuums und der Gesellschaft), Universität Rostock, Germany.; ^3^School of Medicine, University College Dublin, Ireland.

**Keywords:** BAT activation, CR mimetics, healthspan, heat stress, cold stress

## Abstract

Climate extremes and rising energy prices present interconnected global health risks. Technical solutions can be supplemented with biomedical approaches to promote healthy longevity in hot and cold conditions. In summer, reducing basal metabolic rate through mild caloric restriction or CR mimetics, such as resveratrol, can potentially be used to lower body temperature. In winter, activating brown adipose tissue (BAT) for non-shivering thermogenesis and improved metabolic health can help adaptation to colder environments. Catechins found in green tea and in other food could be alternatives to drugs for these purposes. This review examines and discusses the biomedical evidence supporting the use of CR mimetics and BAT activators for health benefits amid increasingly extreme temperatures.

## 1. Introduction

With the ongoing climate change, extreme weather conditions, like storms and heat waves, are becoming more common on a global scale [1]. Such periods of extreme heat pose a serious threat to all forms of farming and agriculture and thus to the aim of feeding an ever-growing population. However, heat waves are also directly impacting the health and wellness of humans. Direct physiological consequences include, for instance, a redistribution of the blood flow towards the skin to increase cooling via sweating and evaporation of fluid [2]. Physical fitness and the capacity to perform manual labor are reduced, with detrimental effects for the economy. Further changes also occur in the renal and cardiac system (increased pump rate) to compensate for the heat stress [3]. Lung damage such as pulmonary edema and acute respiratory distress syndrome can result from such conditions [4], a situation which is exacerbated in people with existing health problems and high risk groups, such as the very young and old or outdoor workers; see also [3] for an in depth description of the health risks posed by heat extremes. Furthermore, a large study investigating almost 65 million deaths estimated that in 2019 almost 1.7 million deaths were attributable to non-optimal temperatures [5].

Heat waves are a problem with global consequences and, ideally, remedies should therefore also be applicable and affordable globally. There are a number of easy strategies that fall into this category. Shifting working times towards mornings and evenings and thus away from the most intensive heat is one option that has been historically practiced in Mediterranean countries. Appropriate clothing and staying in the shade are other alternatives. Yet, the International Energy Agency reports a growing demand for air conditioners (AC) to keep cool, a strategy that already accounts for nearly 20% of all electricity used in buildings around the world (www.iea.org/reports/the-future-of-cooling). Since most homes in developing countries so far do not possess AC this trend is expected to continue in the next decades. Clearly, this approach is not sustainable, both due to costs (especially for the poorest countries) and the fact that it drives further global warming and heat waves [1, 6]. On the other hand, we note the rise in energy prices in recent times, making heating in winter increasingly expensive.

Facing climate extremes and energy costs, we like to suggest here that technical solutions may be complemented by biomedical approaches that may simultaneously foster healthy longevity. During hot summers, a lowering of the basal metabolic rate would be useful to reduce thermogenesis and to slightly lower body temperature, suggesting mild caloric restriction (CR) or, more conveniently, intake of CR mimetics. Also, a diet rich in electrolytes can counteract their loss by sweating [7]. In winter, when high energy prices might prevent sufficient heating, brown adipose tissue (BAT) could be an interesting target for intervention. In young as well as adult humans, beige and brown adipose tissue is involved in non-shivering thermogenesis. We propose that BAT activation could not only adapt people to colder rooms but also yield better metabolic health. While some drugs exist for BAT activation, recent research suggests a safer approach is through the use of natural (food) compounds for BAT activation. In summary, we here review the biomedical evidence that CR (and CR mimetics) and BAT activators may provide small yet measurable wellness effects in times of increasingly extreme temperatures, while improving health at the same time.

## 2. Caloric Restriction (CR) and mimetics

Caloric restriction has a long history showing its beneficial effects on health and lifespan in various species. Evidence goes back at least to 1935 when it was shown that the mean and maximum lifespan of rats kept under caloric restriction, but without malnutrition, was significantly increased compared to a control group [8]. Since then, lifespan extension via CR has been repeatedly confirmed in flies, worms, mice and monkeys [9-13]. Importantly, CR also increases healthspan, i.e. the fraction of disease free lifespan, which is an important aspect considering application to humans [14] by improving various clinical parameters [15] and reducing the incidence of various diseases like cancer, cardiovascular events and degenerative disorders [16-18].

Various recent studies have analyzed the effects of CR on body temperature in humans. Overall, their findings agree that caloric restriction is causing a reduction in body temperature, although not all studies reported statistically significant results. Two studies in particular, one by Heilbronn et al. [19] and one by Soare et al. [20] have reported significant reductions in core body temperature following CR.

The first [19] involved 48 overweight, nonobese (BMI 25 to <30) men and women, allocated to a weight maintenance diet (control group), to 25% CR, to 12.5% CR plus a structured exercise program designed to induce a 12.5% increase in energy expenditure (CREX), or to a very low-calorie diet (890 kcal/d until 15% weight reduction, followed by a weight maintenance diet). The authors observed significant reductions in core temperature in the CR group by 0.2°C, and by 0.3°C in the combined caloric restriction and exercise (CREX) group. Soare et al. made similar observations in their case-control study. The study involved 24 men and women (mean age 54 years) consuming a CR diet, comparing them to 24 age- and sex-matched sedentary volunteers on a Western diet (WD) and 24 body fat-matched, exercise-trained (EX) volunteers. The mean 24-hour core body temperature in the CR group was 36.64°C, which was significantly lower than that of the WD group (36.83°C) and the EX-group (36.86°C). In the CALERIE 2 study, a two-year, multicenter, phase 2 randomized controlled trial, 218 nonobese individuals aged between 21-51 years were subjected to either 25% CR or an ad libitum (AL) diet [21]. Although the CR group reached statistically significant reductions in body temperature (-0.05°C) compared to baseline, the reduction compared to the AL group was not significant, because body temperature also declined in the AL group for unknown reasons.

The most likely underlying mechanism of these observed changes in body temperature is adaptive thermogenesis, which refers to the downregulation of the metabolic rate in response to the decrease in caloric intake. Adaptive thermogenesis has also been observed in monkeys, even after correction for lean body mass [22]. Furthermore, a reduction of basal metabolic rate was a long-term effect observed in rhesus monkeys subjected to moderate CR for more than ten years [23]. The same phenomenon can also be seen in mice or rats under CR [24, 25].

Metabolic rate reduction makes sense since it is a strategy to reduce energy expenditure in situations of food shortage. Although endotherms tend to maintain a constant body temperature by heat generation and dissipation, a lowering of the metabolic rate also leads to a lowering of body temperature. This effect has been found in rodents, non-human primates and humans (as discussed above) [19, 20, 26-29], with it being argued that this reduction in body temperature is causally involved in the life-extending effects of CR [30]. This view is supported by work on transgenic mice with an artificially reduced body temperature. Such mice show an increased median lifespan, even without CR [31].

**Figure 1.**
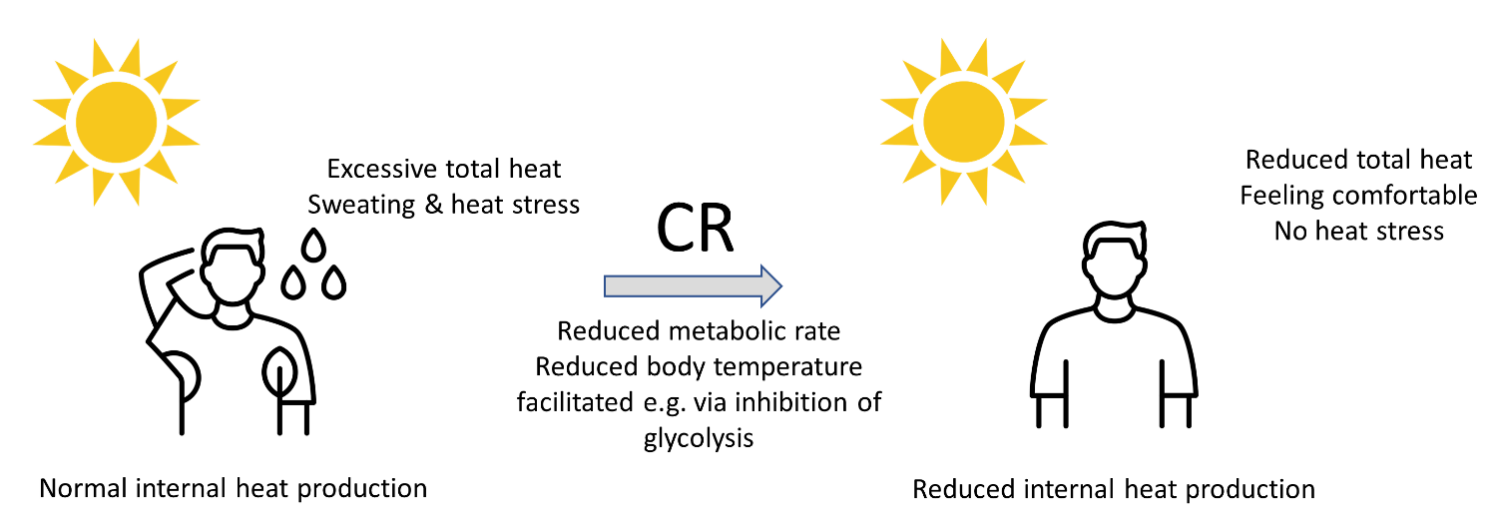
**During heat waves the normal internal heat production together with external heat input led to sweating and heat stress**. Caloric restriction reduces the basal metabolic rate and internal heat production, reducing total heat input leading to more comfortable feeling and less or no heat stress.

We propose that a reduction of the metabolic rate could also be a useful strategy during periods of increased environmental heat. The ensuing reduction of body temperature would normally induce a feeling of being cold [28], but the high temperatures provide additional external heating for the body and consequently counteract the feeling of cold (Fig 1). Without such a reduction of metabolic rate, however, increased environmental temperatures would lead to sweating and heat stress.

A major obstacle regarding the widespread application of CR to humans is of course compliance, and several studies of CR in humans found that hunger is a major limiting factor in this regard [28]. A way forward may be the use of appetite suppressants or CR mimetics. For instance, Semaglutide, sold under the names Wegovy and Ozempic, is an injectable drug originally developed to treat diabetes, but was approved by the Food and Drug Administration (FDA) in 2021 for chronic weight management. When CR mimetics were originally proposed [32] the idea was to identify substances that mimic the beneficial effects of CR (hormonal, physiological, reduction of age-related diseases and life extension) without the need to actually reduce caloric input.

### 2.1 CR mimetics (CRM)

Substances with the potential to mimic important aspects of caloric restriction might have various molecular targets and can be natural food ingredients or synthetic supplements. CRMs are not a well-defined chemical class of compounds, but a loose umbrella term for substances with properties mirroring some of the many CR effects. Inhibitors of glycolysis, intestinal fat and carbohydrate metabolism are obvious candidates, but activators of the AMPK pathway and sirtuins are also possible candidates, in addition to inhibitors of the mTOR pathway and polyamines [33, 34].

The search for glycolytic inhibitors has mostly concentrated on the first two reactions of glycolysis, catalyzed by hexokinase and glucose-6-phosphate isomerase. 2-deoxy-D-glucose (2DG) inhibits the second step of glycolysis and is one of the first and most thoroughly studied CR mimetics. Further, it has been shown to reduce plasma insulin and body temperature [32]. Interestingly, it also led to a lifespan extension in worms [35], reproducing another hallmark of CR. Unfortunately, long term studies showed that 2DG led to cardiac vacuolization and increased mortality in rats [36] making its practical use as a CR mimetic in humans unlikely. The long-term toxicity of 2DG highlights a general problem of synthetic compounds. While studies to uncover such effects can in principle be performed in animal models, there is always the risk that a substance might behave differently in humans. Thus, while not every putative CR mimetic is derived from food, those which are, may be considered to be safer than those which are not. This is because, historically, food derived mimetics have been consumed regularly for a long time without noting severe side effects. As such we will concentrate on CRMs found in food compounds, acknowledging however, that isolated food compounds may still pose safety risks and that there are many drugs with a long history of usage without major side effects.

D-Allulose and D-Glucosamine, also inhibitors of glycolysis, are examples of such naturally occurring CR mimetics. Small amounts of D-Allulose are found in wheat and it has been shown to increase the lifespan of nematodes [37]. D-Glucosamine (found in cartilage and chitin of shellfish) also extends lifespan in animal models (nematodes and mice) [38, 39], induces mitochondrial biogenesis and lowers blood glucose levels [38]. D-Glucosamine has been extensively studied, mainly as treatment of osteoarthritis, and it mimics several of the effects of CR [14].

Polyphenols are a chemically diverse group of molecules that have been proposed as promising candidates of CR mimetics [40]. For instance, resveratrol, curcumin, catechin and quercetin belong to this group. Various studies found that resveratrol reduces waist circumference or body weight [41-44], supporting the idea that resveratrol can act as a CR mimetic. However, while there is evidence that administration of resveratrol in diabetes patients shows CR-like changes such as AMPK and SIRT1 activation [45], others found no changes in markers of CR in postmenopausal women [46]. Interestingly, similarly to CR, supplementation with resveratrol leads to a reduction of resting metabolic rate [47] in human and a small but significant reduction of body temperature [48] in monkeys. Another polyphenol that has been studied intensively is quercetin. Various meta-analyses found an effect on plasma lipid profiles and related health parameters [49-52], but currently evidence for an effect on body weight or body temperature is lacking [53]. Much more work exists regarding CR mimetics and the reader is referred to the following reviews for more detailed information [14, 34, 54, 55].

## 3. Brown Adipose Tissue (BAT)

It has long been known that brown adipose tissue (BAT) is a major source of non-shivering thermogenesis in rodents [56]. In contrast to white adipose tissue (WAT), brown adipocytes show high expression of uncoupling protein 1 (UCP1), include multiple small lipid vacuoles and possess a substantial number of mitochondria [57]. UCP1 is a transmembrane protein of the inner mitochondrial membrane, allowing protons to flow into the organelles bypassing ATP-synthase, thereby dissipating membrane potential and generating heat instead of ATP [58]. The presence of BAT in adult humans was confirmed some years ago [59-62] with the use of positron emission tomography (PET). BAT not only affects the energy balance equation (EE) of input and expenditure, but is also involved in the uptake of lipids and glucose from the blood [57, 63, 64]. Therefore, it has been proposed as a therapeutic target to treat obesity, metabolic syndrome, hyperglycemia and dyslipidemia [65-69].

Although the energy dissipation per gram of BAT (via glucose and fatty acid uptake) is higher than that of WAT or muscle cells [65], the overall contribution to the energy balance equation is smaller since adult humans possess only 10-300 grams of BAT [61, 70-73]. Furthermore, the amount of BAT declines with age and body mass index (BMI) [62, 68, 74-76], which may be counteracted by increasing its mass or stimulating its activity. An efficient inducer of BAT activity is cold exposure [71, 72, 77-81] and this is also the method that has been used in most human studies [82-84]. Because of the accompanying discomfort it is unlikely that this strategy will be followed voluntarily by a large fraction of the population, despite the mentioned beneficial effects. Since BAT activation is controlled by sympathetic neurons, activation of β-adrenergic receptors (AR) [57, 65] through pharmacological compounds like mirabegron has been studied [85, 86]. However, the observed side effects such as increased heart rate and high blood pressure severely restrict their therapeutic application [65, 68].

**Figure 2.**
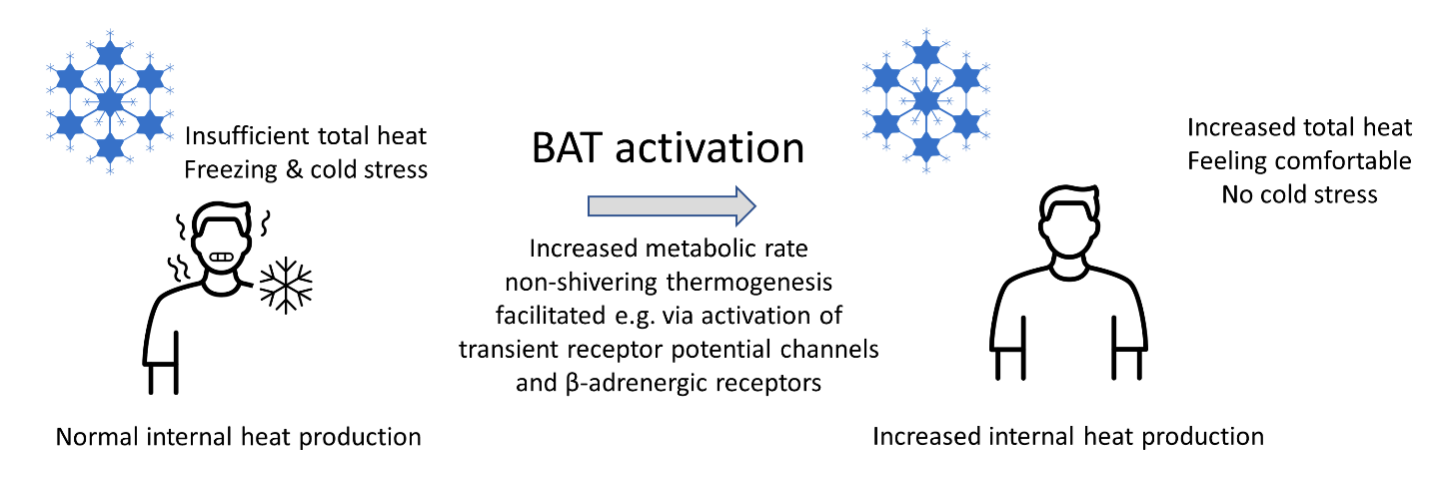
**During winter the normal internal heat production together with insufficient heating can lead to freezing and cold stress**. Activation of brown adipose tissue (BAT) increases non-shivering thermogenesis and total heat output leading to more comfortable feeling and less or no cold stress.

Another approach is to increase the mass of BAT, which would also be relevant for older people and those with a high BMI. ‘Browning’ of WAT into beige adipocytes is a way to achieve this. Although beige and brown adipocytes have distinct gene expression patterns [68], beige adipocytes are also capable of thermogenesis [87, 88]. Several browning inducers have been identified such as fibroblast growth factor 21 and several members of the TGF-β superfamily [89-94]. Interestingly, food ingredients like capsaicin, capsinoids and tea catechin can also induce browning [66, 95, 96].

We propose that BAT activation is suited to help during times of high heating costs. One of the potential concerns regarding BAT activation is hyperthermia [65, 68], since the increased heat production, in the case of normal environmental temperatures, could lead to an undesirable increase of body temperature. If, however, room heating is reduced, BAT activation would mitigate cold stress, and merely maintain body temperature at normal levels (Fig 2). To eliminate further unwanted side effects, we also suggest avoiding pharmacological BAT activation and instead make use of the observation that food ingredients can also achieve the same effect. Several studies have investigated the effect of various nutritional components on BAT activation. However, we will concentrate here on the most popular and promising interventions and refer the interested reader to existing reviews regarding this topic for further details [66, 67, 69, 97-99]. BAT thermogenesis is also induced shortly after a single meal, which is known as diet-induced thermogenesis (DIT) [57, 66], but here we are interested in the more long-term effects of non-caloric food ingredients.

### 3.1 Capsaicin and Capsinoids

Capsaicin and capsinoids are popular thermogenesis candidates, which both bind to TRPV1, a member of the family of transient receptor potential (TRP) channels. Activity of these receptors then activates β-adrenergic receptors (β-AR) further downstream. Although both substances have similar chemical structures, the pungency of capsinoids is around 1000 times less than that of capsaicin, which is found in chili peppers [66].

Oral administration of these substances in studies involving mice and rats has shown that by activating TRPV1, BAT activity and whole-body EE is increased [100-102]. Such an increase of EE after a single capsinoids consumption was also found in humans [103]. Interestingly, that increase was only observed in participants with active BAT, indicating a causative role of BAT for the induced thermogenic effects. Since there is great variability in the human population regarding the amount of active BAT, it is important that administration of capsinoids over a longer period (6 weeks) can activate BAT and increase its amount in humans [95, 104, 105]. Since the daily ingestion of several milligrams of capsinoids has no serious negative side-effects [95, 105-107] it appears to be safe for human consumption.

A recent systematic review covering publications from 2007 to 2018 [67] found six studies investigating the effects of capsinoids in humans [95, 103, 104, 108, 109]. Most studies were conducted in young, non-obese males using a daily dose of 9 mg over several weeks. All but one showed that capsinoids could activate BAT based on cold induced thermogenesis (CIT), resting metabolic rate (RMR) or PET/CT scan. These findings are promising, but further work is needed since all studies have a small sample size (3-24 participants) and not all followed a double-blind, randomized, placebo-controlled design.

The family of TRP receptors are not cold sensors, but are involved in sensing hot temperatures. While TRPV1 is activated above 43°C, other members like TRPM8 are active at lower values around 17-25°C [66]. Interestingly, there are also compounds like menthol (found e.g., in the oils of peppermint and spearmint) that function as TRPM8 antagonists and consequently cause a sensation of coolness. Because of the lower temperature range of TRPM8, activating this receptor should mimic the effects of a mild cold exposure and this has also been confirmed in several mouse studies [110, 111] although its effects in humans are unknown. However, the combination of menthol with capsaicin and capsinoids might be an interesting strategy to mitigate the pungent feeling that cannot be tolerated by some people.

### 3.2 Catechins and Quercetin

Catechins are found in copious amounts in green tea and belong to the class of polyphenols, which display antiobesity, anticarcinogenic, antibacterial and antiinflammatory properties [112-114]. Over the last twenty years several human studies have shown that catechin intake leads to a short-term thermogenic effect and increase in EE [115-118]. There exists also a causal link to BAT activation since catechin consumption only increased EE and CIT in participants with high levels of BAT [96]. Despite the different chemical structures, it seems that catechins, like capsinoids, stimulate BAT through the TRP pathway, although further work is required to clarify this point [66]. Catechins are often administered together with caffeine, as in two studies that went on for 5 and 12 weeks respectively [96, 105]. Both studies found a significant increase in BAT density, CIT and resting metabolic rate. Since the placebo group of one study [105] also contained caffeine, but did not show the response of the active group, it seems the observed BAT activation is linked more to the catechins instead of the caffeine.

The effects of quercetin, a flavonoid with similar chemical structure as catechin have often been studied in mice with respect to anti-obesity effects [119-122]. However, more recently connections between quercetin and BAT have also become apparent, since it upregulated markers of WAT browning [123-125] and UCP1 mRNA [126] in mice. In humans the situation is not as clear, since a meta-analysis of 9 randomized controlled trials (RCT) of quercetin intake covering a range of doses and intervention times did not find beneficial effects on body weight [53]. However, there exist also several RCTs showing that quercetin does reduce blood pressure, blood lipids and waist circumference [127-129]. Polyphenols such as catechins and polyphenol-rich diets in general also improve flow-mediated dilation, contributing to lower blood pressure, and thus counteracting the increase in blood pressure due to colder temperatures [130].

### 3.3 Others

A plethora of additional food compounds have been tested for BAT activation and control of body weight, which we do not cover here due to space constraints and because the evidence is often quite weak. Among those are for example pterostilbene (the dimethylated derivative of resveratrol), luteolin, phytoestrogens, ephedrine, xanthigen, seeds of *A. melegueta*, linoleic acid, casein, curcumin, garlic powder and ginger extracts, amongst others. For more details on those substances the reader is referred to [66, 67, 69, 99, 131, 132].

## 4. Discussion and Summary

Heat waves and rising energy prices are two emerging challenges of recent years. The physiological consequences of extreme heat include changes in blood flow, reduced physical fitness and labor capacity, renal and cardiac system changes, and increased risk of lung damage. Moreover, heat waves have been associated with a significant number of deaths. Given the global nature of heat wave problems, it is important to develop interventions that are applicable and affordable worldwide. Additionally, increasing energy costs may prevent adequate heating during colder seasons, which can also have negative consequences for well-being and health, in particular in vulnerable populations.

We propose here a biomedical approach to mitigate these problems, which might have the added benefit to also improve health. We hypothesize that with the help of CR mimetics basal metabolic rate and thus thermogenesis could be reduced during periods of high temperatures (reducing heat stress) and that the activation of BAT during wintertime could increase thermogenesis to avoid cold stress. There is good evidence that both effects can be achieved by natural ingredients from food (e.g., resveratrol or catechins), providing a safe and affordable route to this approach.

Unfortunately, our current knowledge about such substances is still limited and the sample sizes of human studies are quite small, often comprising less than two dozen participants [67]. Clearly it is necessary to improve this situation to obtain reliable estimates of effect sizes and to uncover possible side effects. More work is also needed to clarify the exact role of polyphenols regarding CR effects and BAT activation since they have been implicated in both. To clarify the situation, it would be important to measure the effect on resting metabolic rate and especially body temperature in future studies, as a simple to measure but still valuable parameter. One possible way is to use smart wearables, which would even provide time resolved data. Currently, a reduction of body temperature has been observed for at least two CR mimetics, 2DG and resveratrol, which is very promising. Unfortunately, 2DG has serious side effects, supporting our suggestion to concentrate on food compounds for safety reasons.

The discussed interventions have potential applicability and value in addressing the challenges posed by climate related temperature extremes. However, there are timing issues to consider, such as the need for preparation before heat waves occur, and the individual commitment required for caloric restriction or BAT activation. Also, effect sizes are quite modest considering any single individual implementing these interventions. Yet, if implemented on a larger scale, the sum of the effects can be expected to be notable, in terms of the use of resources as well as the improvements in health. Socioeconomic factors, including the affordability and accessibility of interventions, need to be taken into account to ensure equitable benefits.

In conclusion, the biomedical interventions of caloric restriction mimetics, as well as the activation of brown adipose tissue, offer approaches to mitigate the negative impacts of extreme temperatures and higher energy costs. These interventions can be globally applicable, although challenges regarding compliance, timing, and socioeconomic factors need to be addressed. Further research and development are necessary to explore the effectiveness, safety, and practical implementation of these interventions on a larger scale.
